# Lupus vulgaris mimicking hemangioma diagnosed 42 years after onset: a case report

**DOI:** 10.1186/s13256-015-0667-8

**Published:** 2015-09-16

**Authors:** Mahizer Yaldız, Teoman Erdem, Bahar Sevimli Dikicier, Fatma Hüsniye Dilek

**Affiliations:** 1 Dermatology Department, SB. Sakarya University Hospital, Esentepe Kampüsü, 54187 Sakarya, Turkey; 2 Pathology Department, SB. Sakarya University Hospital, Esentepe Kampüsü, 54187 Sakarya, Turkey

## Abstract

**Introduction:**

Lupus vulgaris is the most common form of cutaneous tuberculosis. It may easily be overlooked if a proper differential diagnosis is omitted.

**Case presentation:**

A 46-year-old Turkish woman presented with a 42-year history of erythamatous plaque on her left arm. Ziehl–Neelsen and periodic acid-Schiff stains did not show any acid-fast bacilli. Culture from a biopsy specimen was negative for *Mycobacterium tuberculosis*. The result of a polymerase chain reaction-based assay for *Mycobacterium* was negative. Histopathologic findings revealed a tuberculoid granuloma containing epithelioid cells, lymphocytes and Langerhans-type giant cells. A diagnosis of lupus vulgaris was made by clinical and histopathologic findings.

**Conclusions:**

The lesion improved after antituberculous therapy, confirming the diagnosis of lupus vulgaris.

## Introduction

Lupus vulgaris (LV) is the most common clinical type of cutaneous tuberculosis (TB) in adults [1, 2]. LV commonly appears on the face [3, 4]. LV may rarely be seen on the extremities [5]. The course of LV may continue for years, if it is left untreated [6]. Here, we report the case of a patient with long-lasting LV mimicking hemangioma.

## Case presentation

A 46-year-old Turkish woman presented to our institute with a red, well-demarcated plaque on her left arm. She said the lesion had appeared when she was 4 years old. A doctor who our patient had visited when the lesion first appeared had said that the lesion was a birthmark. After that, our patient did not see any doctor with regard to this lesion. However, over the last 2 years, the lesion had started to enlarge, and she consulted a doctor again; this time she was told that it was a harmless vascular lesion, described as a hemangioma.

**Fig. 1:**
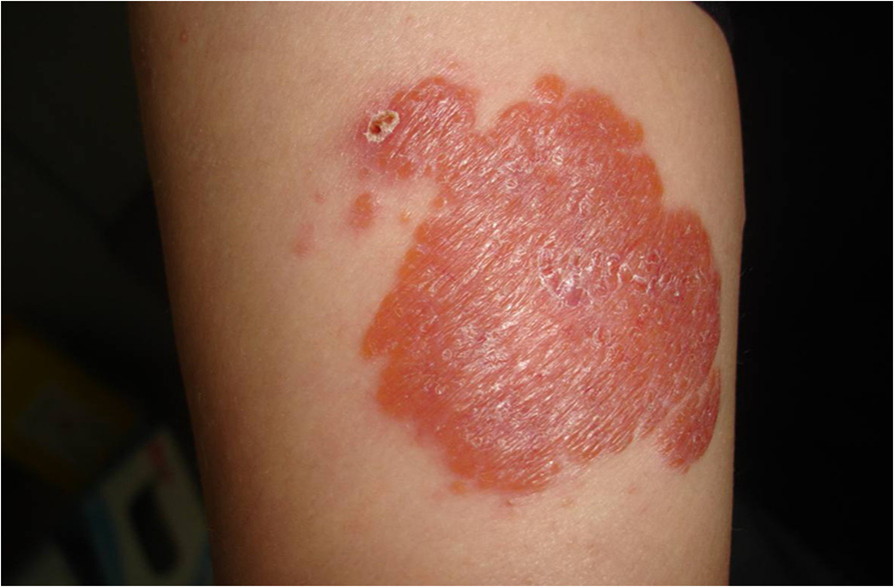
Erythematous, nontender, well-defined plaque and satellite papules present over the left medial arm

**Fig. 2:**
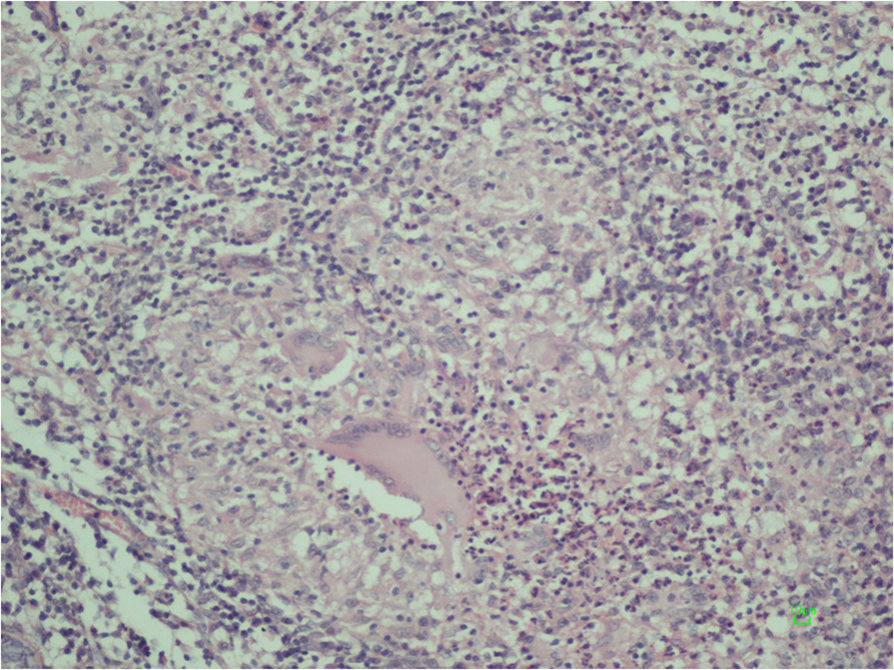
Granulomatous infiltration in the dermis with lymphocytes and Langerhans-like giant cells (hematoxylin and eosin original magnification ×100)

A dermatological examination revealed an erythematous, nontender, 5×7cm in size, well-defined plaque present over the medial part of her left arm. Some erythematous satellite papules, 0.5×1cm in size were scattered around the main lesion (Fig. 1). The lesion did appear to resemble a hemangioma at first sight. Diascopy of the lesions revealed an apple-jelly appearance. There was no lymphadenopathy. A systemic examination was normal. No bacille Calmette-Guérin (BCG) scar was visible. An incisional biopsy of the plaques showed the formation of a tuberculoid granuloma composed of epithelioid cells, lymphocytes, and Langerhans-like giant cells located in the upper dermis (Fig. 2). A Mantoux test was positive with an induration of 6mm after 48 hours.

Laboratory tests showed a normal blood count. Sputum, stool and urine cultures were negative. The results of venereal disease research laboratory (VDRL) and human immunodeficiency virus (HIV) tests were negative. Fungal and standard bacterial cultures from the skin biopsy were negative. Ziehl-Neelsen and periodic acid-Schiff stains did not show any acid-fast bacilli. Culture from the biopsy specimen was negative for *Mycobacterium tuberculosis*. A real-time polymerase chain reaction (PCR)-based assay of the biopsy specimen gave a negative result for *Mycobacterium*. Chest radiography and an abdominal ultrasound did not show any pathologic finding. Underlying bone and joint disease was excluded by radiography. Lupus vulgaris was diagnosed in our patient on the basis of the clinical and histopathologic findings.

**Fig. 3:**
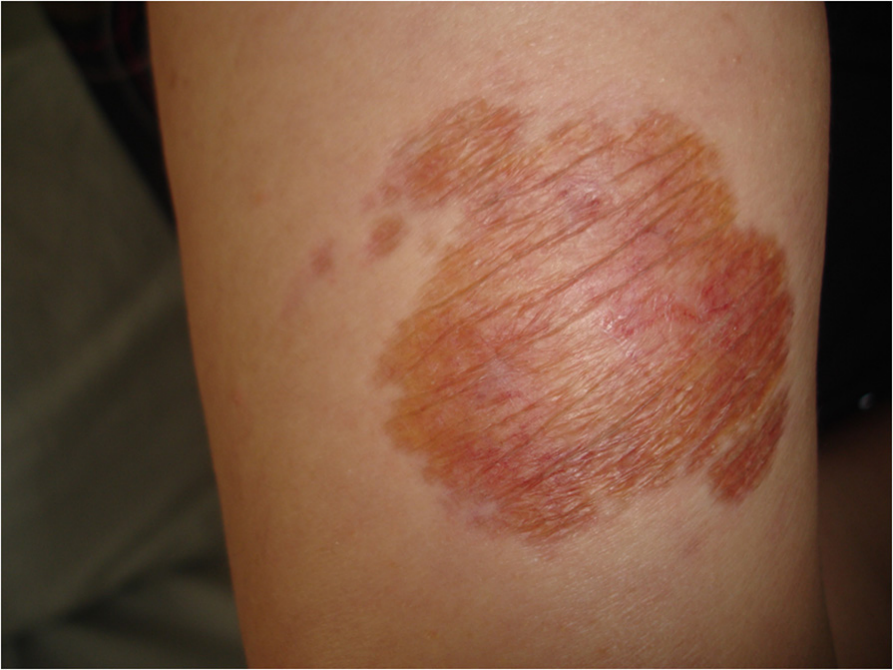
Atrophy and postinflammatory pigmentation after the treatment

Our patient was treated with isoniazid (5mg/kg), rifampin (10mg/kg), ethambutol (25mg/kg), and pyrazinamide (15mg/kg) daily for 2 months, followed by isoniazid and rifampin for 7 months. The cutaneous lesions started to regress within 3 months and had completely healed with atrophic scarring and postinflammatory hyperpigmentation after 9 months (Fig. 3).

## Discussion

Lupus vulgaris is generally regarded as a benign, chronic and progressive form of cutaneous tuberculosis that may be associated with tuberculosis of other organs. LV usually originates from an endogenous source of tuberculosis and is spread hematogenously, lymphatically, or by contagious extension [1, 7]. Less commonly, it is acquired exogenously following primary inoculation tuberculosis or BCG vaccination [8]. No endogenous source for tuberculosis was found, so we concluded that primary inoculation was the case for this patient.

LV is characterized by a macule or papule, with a brownish-red color and soft consistency that form larger plaques by peripheral enlargement and coalescence [2, 9].

The lesions of LV are usually located on the head and neck area [1, 3, 10]. LV is rarely seen on arms and legs; those located on the extremities usually occur by reinoculation [2].

Diagnosing LV may be a formidable task. Various diagnostic methods, including culture, Ziehl–Neelsen staining and PCR may be negative in LV, because of the scarcity of the bacilli within the lesional tissue [11, 12]. In conclusion, the diagnosis usually relies on typical clinical and histologic findings, a positive purified protein derivative (PPD) test and a favorable response to antituberculous therapy [1, 13]. In our patient, the diagnosis of LV was based on typical clinical and histologic findings and excellent response to specific antituberculous therapy. The diagnosis of cutaneous tuberculosis is easily made if one considers it in the differential diagnosis, particularly in those patients with a history of tuberculosis and a suggestive clinical presentation; otherwise, it is easily missed.

The differential diagnosis for our patient included sarcoidosis, other cutaneous tuberculosis types such as tuberculosis cutis verrucosa, scrofuloderma, metastatic tuberculous abscess, necrobiotic xanthogranuloma, leishmaniasis, pseudolymphoma and hemangioma.

## Conclusions

Tuberculosis is still an important problem in underdeveloped and developing countries due to the poor hygiene conditions and low socioeconomic level. Physicians need to be aware of the diagnosis and treatment of lupus vulgaris and other forms of cutaneous tuberculosis.

## Consent

Written informed consent was obtained from the patient for publication of this case report and any accompanying images. A copy of the written consent is available for review by the Editor-in-Chief of this journal.

## Abbreviations

BCG: bacille Calmette-Guérin
HIV: human immunodeficiency virus
LV: lupus vulgaris
PCR: polymerase chain reaction
PPD: purified protein derivative
TB: tuberculosis
VDRL: venereal disease research laboratory
